# Specific versus non-specific hospital-based spine diagnoses in children and adolescents: a nationwide register-based cohort study

**DOI:** 10.1186/s12998-026-00661-z

**Published:** 2026-07-01

**Authors:** Freja Gomez Overgaard, Casper Nim, Laura Montgomery

**Affiliations:** 1 Medical Spinal Research Unit, Spine Centre of Southern Denmark, University Hospital of Southern Denmark, Kolding, Denmark; 2 Department of Regional Health Research, University of Southern Denmark, Odense, Denmark; 3 Department of Chiropractic, Faculty of Medicine, Health and Human Science, Macquarie University, 75 Talavera Road, Macquarie Park, Sydney, NSW 2113 Australia; 4 School of Health Sciences, Faculty of Medicine and Health, The University of Sydney, Sydney, Australia

**Keywords:** Back pain, Adolescent, Child, Hospital records, Diagnosis, Epidemiology, Pediatrics

## Abstract

**Background:**

Paediatric spine pain is common, yet little is known about the influence of patient characteristics on diagnostic classification in hospital settings.

**Objective:**

Describe diagnostic code variation by age, sex, parental spine diagnoses, and child comorbidity burden.

**Methods:**

Nationwide historical (2007–2022) register-based cohort study including Danish 0-17-year-olds with a hospital-based spine-related diagnosis. Diagnoses were classified as specific or non-specific by ICD-10 codes. Patient characteristics were examined descriptively and with logistic regression.

**Results:**

Of 49,596 children, 63% received a non-specific diagnosis. Non-specific diagnoses were more common among older children (OR ~ 0.22–0.27), children with parental spine-related diagnoses (OR = 0.77), male children (OR = 1.07), or children with lower comorbidity burden (OR ~ 1.09–1.13).

**Conclusions:**

Diagnostic classification of paediatric spinal pain varies systematically by patient characteristics. Most children receive non-specific diagnoses, underscoring the need for the development of appropriate care pathways including greater use of primary care for non-specific spinal pain.

**Supplementary Information:**

The online version contains supplementary material available at https://doi.org/10.1186/s12998-026-00661-z.

## Introduction

Spinal pain in children and adolescents (hereinafter, referred to as *children*) is recognised as a common and important musculoskeletal problem, with up to 30% of school-aged children reporting spinal pain within a year [1, 2]. One fifth experience recurrent or persistent spinal pain across adolescence [3–5], which increases their risk of ongoing spinal pain in adulthood [6, 7].

Historically, paediatric spinal pain was considered rare and primarily indicative of severe underlying pathology [8]. In contemporary paediatric musculoskeletal practice, diagnosis is often driven by functional disturbances such as impaired movement, altered biomechanical patterns, or activity-related limitations rather than by identifiable structural pathology [8], consistent with the International Classification of Functioning, Disability and Health framework [9]. This has been reflected in real-world evidence from Denmark, which showed stable overall incidence of spinal pain but a shift in diagnostic composition. Structural diagnoses (e.g., M41 scoliosis) and whiplash-related diagnoses (e.g., S13.4 sprain and strain of cervical spine) declined, whereas non-specific diagnoses (e.g., M54.5 low back pain not otherwise specified) increased [10].

This shift in Danish diagnostic coding may reflect a broader move toward a biopsychosocial understanding of spinal pain, similar to trends observed in adult populations [11]. Nevertheless, a substantial proportion of children continue to receive specific diagnoses, such as scoliosis [10] or radiculopathy [12], which remain essential for appropriate clinical management in selected cases. The increasing use of non-specific diagnoses suggests a selective, rather than uniform, change in diagnostic practice. This raises important questions about when, why, and for whom specific diagnoses are assigned.

In Denmark, children with spine-related conditions may enter hospital care through referral from general practitioners, chiropractors, and other hospital departments, as well as through self-initiated emergency or acute care services. Hospital services are tax-funded and free at the point of use, whereas some primary care services, including chiropractic care, involve patient co-payment. Consequently, hospital-based spine diagnoses likely represent a selected subgroup of children with spinal pain conditions rather than all children presenting with spinal pain in the community. The factors influencing referral to hospital care are likely multifactorial and may include clinical presentation, referral practices, and family healthcare-seeking behaviour.

Understanding whether diagnosis type varies systematically by patient characteristics may provide insight into true clinical differences and the role of diagnostic uncertainty in paediatric practice [13]. Some children, for example younger children, females, or those with greater comorbidities may be more likely to receive specific diagnoses, potentially reflecting biological vulnerability [14–16]. Or others, such as those with parents affected by spinal disorders, may be more likely to receive specific diagnoses due to parental concern influencing consultations, symptom reporting, and clinicians’ thresholds for attributing pain to a defined pathology [17, 18].

The aims of this study were to; (1) quantify the proportion of children receiving specific versus non-specific hospital-based spine diagnoses in Denmark across time, and (2) to describe how diagnostic classification varied according to age, sex, parental history of spine-related diagnoses, and comorbidity burden.

## Methods

### Study design

We conducted a nationwide, register-based historical cohort study using linked data from multiple Danish health and population registers.

This study is reported in accordance with the Strengthening the Reporting of Observational Studies in Epidemiology (STROBE) statement [19] and the REporting of studies Conducted using Observational Routinely-collected health Data (RECORD) guidelines [20].

### Data sources

The Danish civil registration system (CRS) has recorded vital demographic information on all Danish residents since 1968, including date of birth, sex, and links across Danish registers via unique Civil Personal Register (CPR) numbers. We used the CRS to obtain sex, age at index date, and to link children to their parents or legal guardians [21].

The Danish National Patient Register (DNPR) contains nationwide information on all hospital contacts for both somatic and psychiatric conditions in Denmark, including inpatient, outpatient, and emergency visits. Recorded information includes dates of contact and diagnostic information coded according to the International Classification of Diseases, 10th revision (ICD-10). We used the DNPR to identify children with spine-related hospital diagnoses, ascertain comorbidity burden prior to the index diagnosis, and identify parental spine-related diagnoses [22]. Data were accessed through Statistics Denmark’s secure research platform, where individual-level register data are available in de-identified form. Diagnoses in the DNPR are recorded by treating clinicians and coded according to ICD-10 for administrative and reimbursement purposes.

### Study population

All Danish children aged 0–17 years were eligible for inclusion. We included children with an index spine-related hospital diagnosis, defined as their first recorded hospital contact with one of the included ICD-10 spine-related codes, between 1 January 2007 and 31 December 2022.

We excluded children with an invalid CPR number or without a registered residence in Denmark at the index date. The study population represents the complete national cohort meeting these criteria; no a priori sample size calculation was performed.

### Outcome variable

Index spine-related diagnoses were identified using ICD-10 codes M40–M54, M96, M99, and selected codes from S13. These codes capture the spectrum of non-fracture musculoskeletal spine conditions managed in hospital settings, including structural, inflammatory, radicular, and post-procedural.

Fracture diagnoses (e.g., S12 cervical fractures and S32 lumbar fractures) were excluded because they represent acute traumatic injuries with clearly defined aetiology, distinct clinical pathways, diagnostic certainty, and management strategies. These conditions differ fundamentally from non-fracture spine pain conditions and fall outside the scope of our study aims. Trauma-related codes without fracture (e.g., selected S13 sprain and strain diagnoses) were retained, as they represent soft-tissue injuries that may present with diagnostic uncertainty and overlap clinically with other spine pain presentations. Non-spine-related S13 codes (e.g., laryngeal injuries) were excluded.

We dichotomised diagnoses into specific and non-specific categories based on ICD-10 code definitions.

Specific diagnoses included conditions with identifiable structural, inflammatory, radicular, post-procedural, or non-fracture traumatic pathology, including spinal deformities and postural disorders (M40–M43), inflammatory and degenerative spondylopathies and disc disorders (M45–M51), radicular conditions (M54.1, M54.3–M54.5), post-procedural disorders (M96), and selected non-fracture trauma diagnoses affecting the cervical spine (S13).

Non-specific diagnoses were defined as spine-related pain or functional conditions without an ICD-10 code specifying a distinct structural, inflammatory, or radicular pathology. This category included regional or unspecified spine pain diagnoses (M53, M54.0, M54.2, M54.6–M54.9) and biomechanical or functional disorders (M99). These classifications reflect ICD-10 diagnostic groupings rather than clinical diagnoses of exclusion and are based on the formal definitions provided in the ICD-10 codebook.

This classification approach is consistent with that used in our previous nationwide registry-based study [10]. A complete list of all ICD-10 codes used to define spine-related diagnoses and their classification is provided in Supplementary Table S1.

### Exposure variables

Age at index diagnosis was categorised into early childhood (0–4 years), mid-childhood (5–9 years), early adolescence (10–13 years), and late adolescence (14–17 years), reflecting common developmental stages in paediatric research [23].

Biological sex was obtained from the CRS and classified as male or female as registered at birth.

Parental spine history was defined as having at least one parent or legal guardian with a recorded hospital-based spine-related diagnosis (using the same ICD-10 codes as for children) prior to the child’s index diagnosis date, or not. This exposure captures parental spine diagnoses recorded before the child’s index diagnosis and reflects familial clustering of spine-related disorders. Parental spinal diagnoses prior to the child’s index date were further categorised according to diagnosis type. Parents, or legal guardian, were categorised as having (1) *no recorded spinal diagnosi*s, (2) *only non-specific spinal diagnoses*, or (3) *any specific spinal diagnosis* based on hospital-recorded ICD-10 spine diagnoses. If either parent, or legal guardian, had a specific spinal diagnosis, the child was classified in the *any specific parental diagnosis* group. Children were otherwise classified according to whether parents had *only non-specific parental spinal diagnoses* or *no recorded parental spinal diagnosis*. Individual-level linkage between children and their parents was performed using the unique CPR number assigned to all Danish residents. This deterministic linkage is considered complete and highly accurate [21].

Comorbidity burden was defined as the number of distinct ICD-10 chapters represented in the DNPR. This chapter-based approach provides a proxy for multisystem disease burden and has been applied in paediatric populations where traditional comorbidity indices may be inadequate [22, 24]. For example, a child with diagnoses in both Chapter V (mental disorders) and Chapter J (respiratory diseases) would be considered to have a broader burden than one with diagnoses from only a single chapter. The burden was categorised as: no comorbidities (0 chapters), few comorbidities (1–2 chapters), and many comorbidities (≥ 3 chapters).

All exposure variables were assessed prior to, or at, the index date to preserve temporal ordering.

### Statistical analysis

We describe the distribution of specific vs. non-specific spine-related diagnoses across age, sex, parental spine history, and comorbidity burden using counts and percentages. *Post-hoc* descriptive analyses were conducted to examine the distribution of specific diagnoses by sex within age groups.

Data cleaning included identification of the first eligible spine-related hospital diagnosis per child (index diagnosis), removal of duplicate records, and exclusion of individuals with invalid or missing CPR numbers. No missing data were identified for the variables included in the analyses after application of the eligibility criteria [21, 22]. Therefore, no imputation procedures were applied.

Logistic regression models were used to describe differences in the odds (odds ratios (ORs) and 95% confidence intervals (CIs)) of receiving a specific versus a non-specific spine diagnosis across patient characteristics. Crude models were fitted for each exposure variable separately (model 1). Standardised models including calendar year (model 2), and age, sex, and calendar year (model 3) were used to examine whether observed differences in odds remained when demographic and temporal factors were standardised in the model. Analyses were conducted using R version 4.5.1 [25].

## Results

### Study population

We identified 49,596 children, 55% female, with an index hospital-based spine-related diagnosis between 2007 and 2022. The age distribution was skewed towards younger children (median 13.1, IQR 9.1–15.6). Nearly one-third (14,732; 29.7%) had at least one parent or legal guardian with a prior hospital-based spine diagnosis, and more than half (26,839; 54.1%) had a comorbidity burden spanning three or more non-spine ICD-10 chapters. Baseline exposures are in Table 1.

**Table 1:** Baseline exposures of children with an index spine-related hospital diagnosis between 2007 and 2022

Exposure	*n* (%)
Total	49,596 (100%)
Sex	
Female	27,310 (55.1%)
Age group	
0–4 years	4,733 (9.5%)
5–9 years	7,374 (14.9%)
10–13 years	12,198 (24.6%)
14–17 years	25,291 (51.0%)
Parental spine-related diagnosis	
Yes	14,732 (29.7%)
Comorbidity burden	
0 chapters	6,551 (13.2%)
1–2 chapters	16,206 (32.7%)
≥ 3 chapters	26,839 (54.1%)

No missing data were identified for included variables.

### Distribution of spine-related diagnosis type

**Fig. 1:**
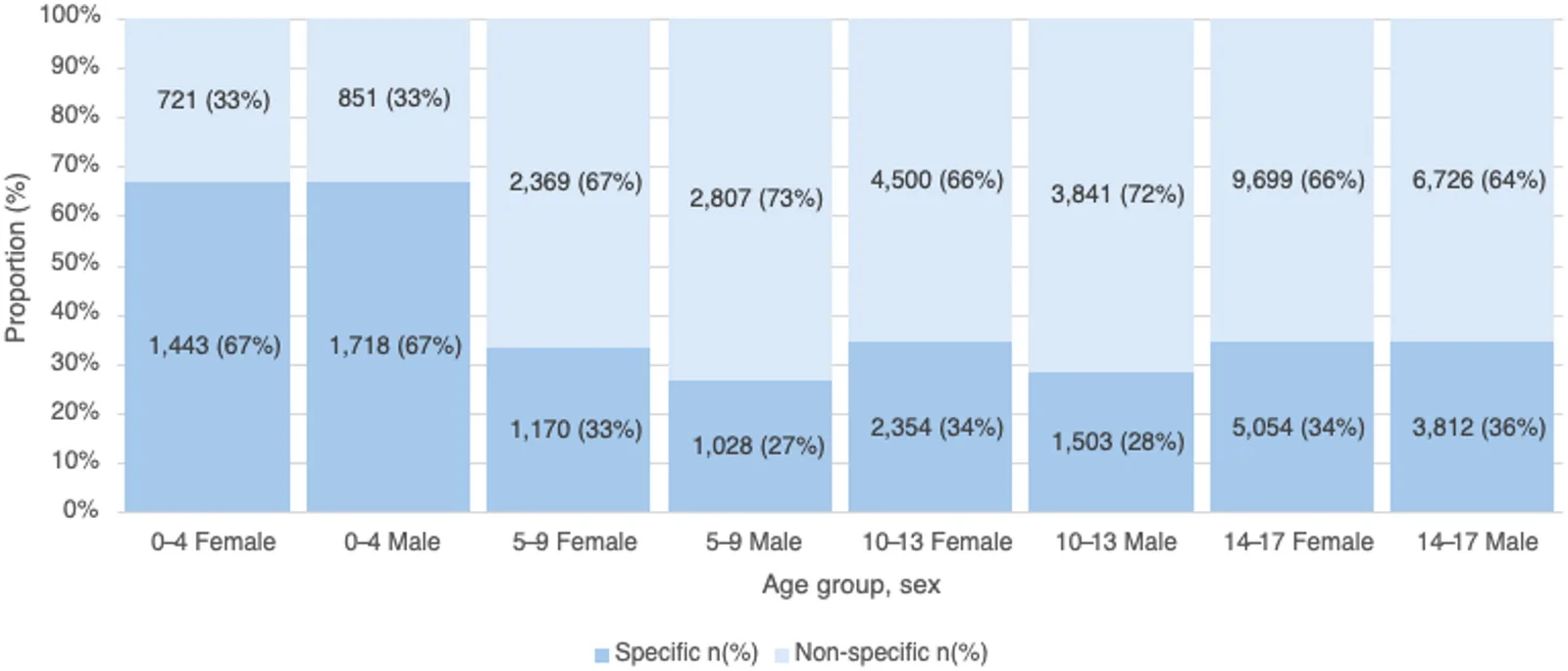
Proportion of specific and non-specific index hospital-based spine-related diagnoses by sex and age group

**Fig. 2:**
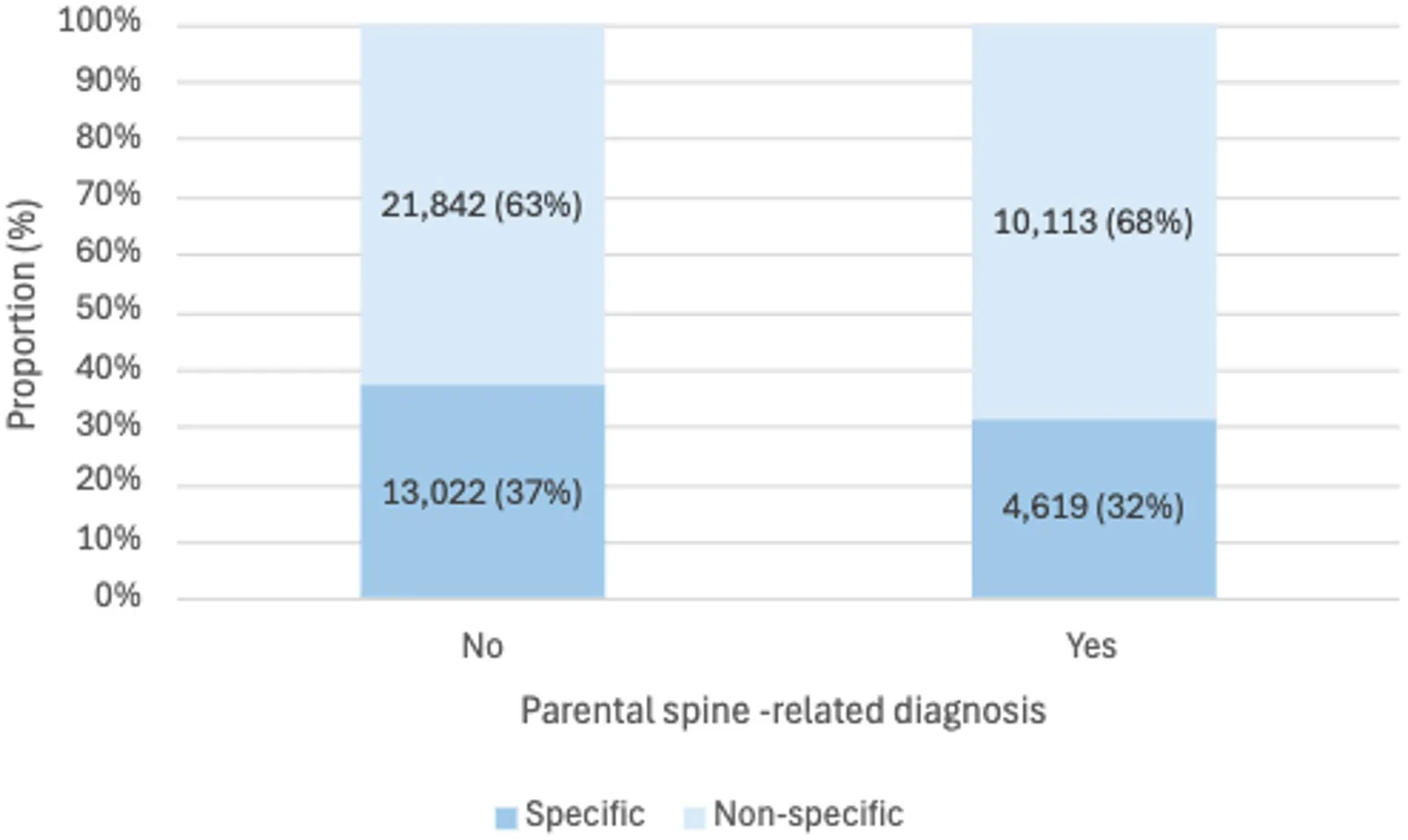
Proportion of specific and non-specific index hospital-based spine-related diagnoses by parental spine-related diagnosis

**Fig. 3:**
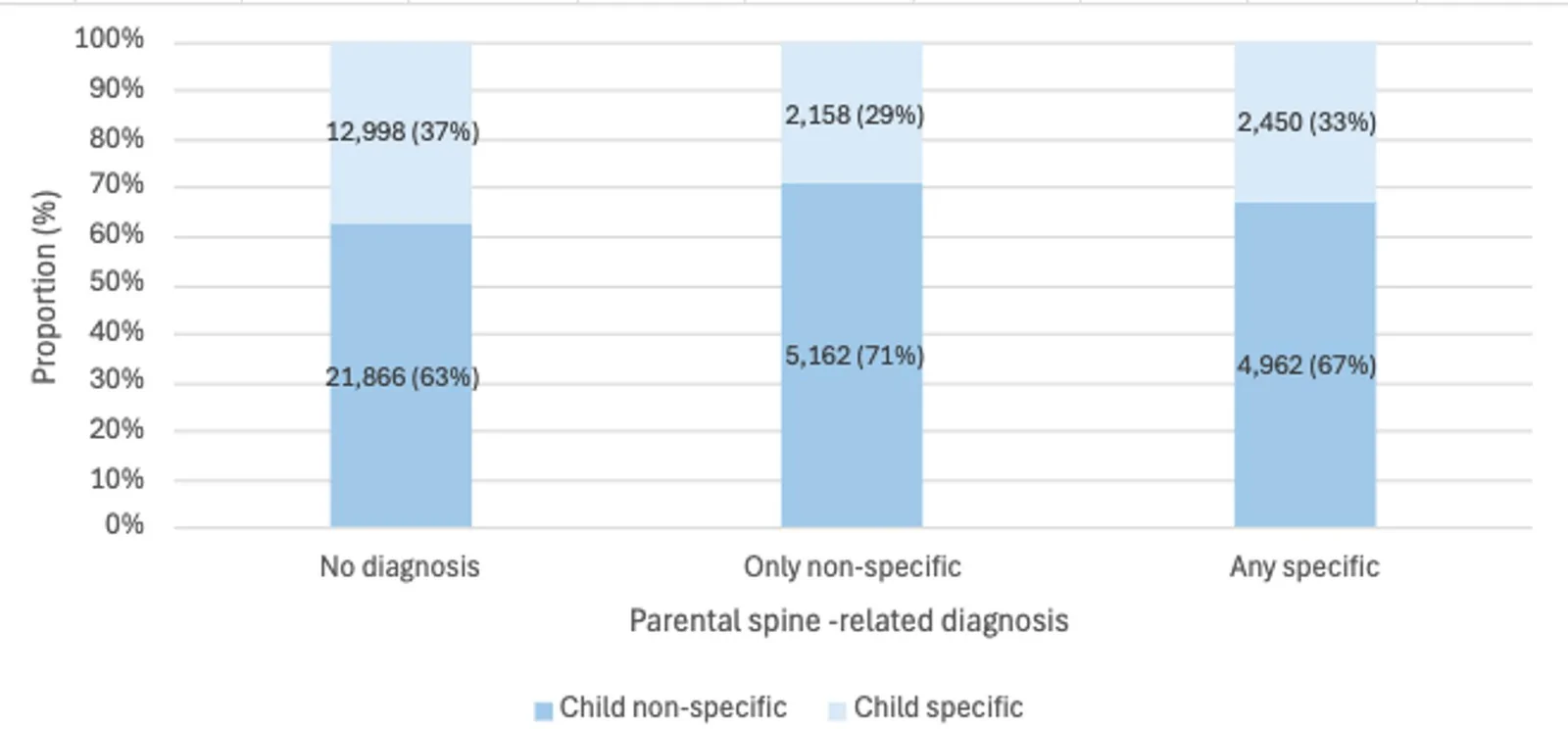
Proportion of specific and non-specific index hospital-based spine-related diagnoses by parental spine-related diagnosis

**Fig. 4:**
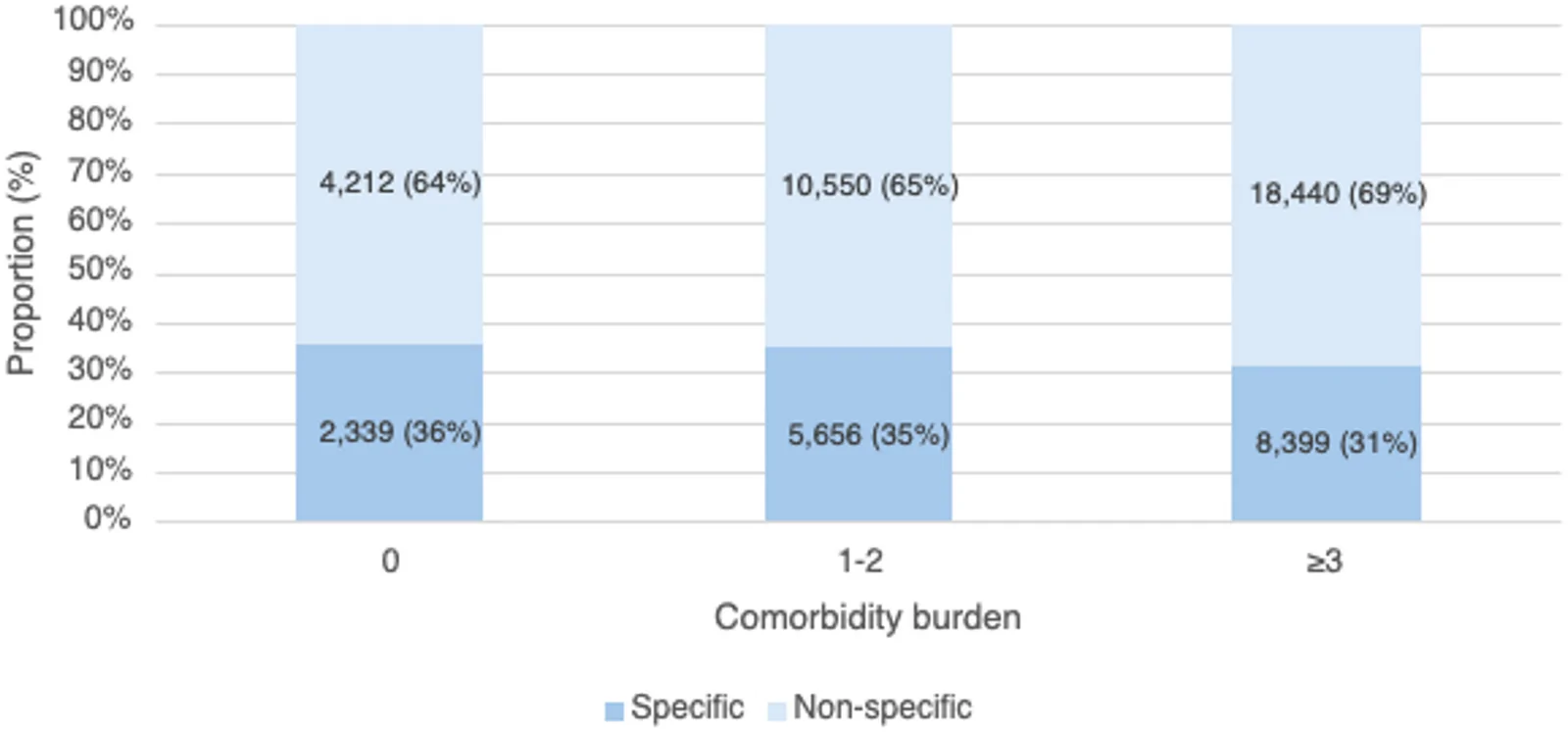
Proportion of specific and non-specific index hospital-based spine-related diagnoses by comorbidity burden

Overall, non-specific spine-related diagnoses (31,955; 63%) were more common than specific diagnoses (17,641; 37%). Specific diagnoses were slightly more common among females. In early childhood (0–4 years), approximately two-thirds of children received a specific diagnosis, whereas in older age groups the majority received non-specific diagnoses. This age-related pattern was similar for females and males (Fig. 1). The distribution of specific vs. non-specific spine-related diagnoses differed minimally according to parental spine-related diagnosis history. Non-specific diagnosis were the most common regardless of parent spine-related diagnosis history, 68.4% (*n* = 10,113) for those with a parent with a hospital-based spine related diagnosis compared to 62.6% (*n* = 21,842) for those without (Fig. 2). The distribution of children’s diagnosis type differed across parental diagnosis groups. Children with only non-specific parental diagnoses more often had non-specific diagnoses themselves (5162; 70.5%), whereas children without parental spinal diagnoses had the highest proportion of specific diagnoses (12,998; 37.3%) (Fig. 3). Children with higher comorbidity burden had a minimally greater proportion of non-specific diagnoses, (18,440; 69% vs. 4,214; 64%) (Fig. 4).

### Regression analysis

Females had slightly higher ORs of receiving a specific diagnosis when standardised for calendar year and age (model 3), see Table 2. The ORs were similar between sexes in the crude estimate (model 1) and when standardised only for calendar year (model 2).

Compared with children aged 0–4 years, all older age groups had substantially lower OR of receiving a specific diagnosis. These estimates were stable across crude and standardised models.

Children with at least one parent or legal guardian with a prior hospital-based spine diagnosis had lower odds of receiving a specific diagnosis than those without, with similar estimates across all models.

Comorbidity burden showed no difference in ORs with diagnosis type in crude estimates (model 1). After standardisation for calendar year (model 2), higher comorbidity burden had higher odds of receiving a specific diagnosis. This estimate was attenuated after further standardisation for age and sex (model 3), though remained positive.

**Table 2:** Odds for receiving a specific versus non-specific index hospital-based spine-related diagnosis

		Model 1 OR (95%CI)	Model 2 OR (95%CI)	Model 3 OR (95%CI)
Sex	*Male*	*Ref*	*Ref*	*Ref*
	Female	1.02 (0.98 to 1.06)	1.02 (0.99 to 1.06)	1.07 (1.03 to 1.11)
Age group	*0–4 years*	*Ref*	*Ref*	*Ref*
	5–9 years	0.21 (0.19 to 0.23)	0.22 (0.20 to 0.23)	0.22 (0.19 to 0.23)
	10–13 years	0.23 (0.21 to 0.25)	0.24 (0.22 to 0.26)	0.24 (0.22 to 0.25)
	14–17 years	0.27 (0.25 to 0.29)	0.28 (0.26 to 0.29)	0.27 (0.26 to 0.29)
Parental spine-related diagnosis	*No*	*Ref*	*Ref*	*Ref*
	Yes	0.76 (0.72 to 0.78)	0.74 (0.71 to 0.77)	0.77 (0.74 to 0.80)
Comorbidity burden	*0 chapters*	*Ref*	*Ref*	*Ref*
	1–2 chapters	0.98 (0.92 to 1.04)	1.17 (1.09 to 1.24)	1.13 (1.06 to 1.21)
	≥ 3 chapters	0.99 (0.94 to 1.05)	1.45 (1.36 to 1.55)	1.09 (1.02 to 1.17)

Model 1; crude. Model 2; standardised for calendar year. Model 3; sex standardised for calendar year and age, age standardised for calendar year and sex, parental spine diagnoses and comorbidity burden standardised for calendar year, age and sex.

In *post-hoc* descriptive analyses, females accounted for a larger proportion of specific diagnoses in the 5–17-year age group (9809 (55.6%) vs. 7832 (44.4%) males), largely driven by scoliosis diagnosis. In contrast, sex differences were smaller in early childhood (1754 vs. 1678 males) (Supplementary Table S2).

## Discussion

In this nationwide historical cohort study of Danish children with hospital-based spine-related diagnoses, we describe how diagnostic classification varies by biological sex, age group, parental hospital-based spine-related diagnosis, and child comorbidity burden.

Across models including calendar year, age, and sex, there were higher ORs of non-specific diagnoses in older children (OR range 0.22–0.27) and in those with one or more parents with a hospital recorded spine-related diagnosis (OR of 0.77), than in younger children or those without parental history. Specific diagnoses had slightly higher ORs in females (OR of 1.07) and in children with higher comorbidity burden (OR range 1.09–1.13), than in males or those with lower comorbidity burden.

Together, these findings indicate that the distribution of diagnostic classifications differed across child characteristics and family context. The largest differences were observed between children aged 0–4 years and older than 4 years. Smaller differences were observed according to parental spine-related diagnosis history, sex and comorbidity burden.

### Interpretation of findings

In our study, age showed the strongest association with diagnostic classification. Relative to 0–4-year-old’s, the ORs of receiving a specific spine-related diagnosis were considerably lower for all other age groups. This indicates a marked shift towards non-specific diagnoses after early childhood. The predominance of non-specific diagnoses in early childhood and in older children and adolescents aligns with previous evidence that non-specific spine pain becomes more common in adolescence [4, 5, 8].

These age-related patterns may relate to developmental and clinical differences across childhood and adolescence. As children mature, musculoskeletal growth, hormonal and neurocognitive changes, and psychosocial and behavioural factors influence pain perception, interpretation, and communication [23]. Although overall sex differences in diagnostic classification were modest, *post-hoc* analyses showed that among older children (5–17 years) with specific diagnoses, females outnumbered males. This pattern is consistent with the higher prevalence and progression risk of adolescent idiopathic scoliosis among females, a condition that commonly requires hospital-based assessment and follow-up.

In contrast, sex differences were less pronounced in early childhood (0–4 years), where diagnostic patterns are more likely driven by developmental and congenital conditions rather than sex-specific risk profiles. In this age group, spinal presentations may be prompted by visual abnormalities or higher levels of child or parent distress, leading clinicians to rely more heavily on physical examination and imaging. In older children and adolescents, where sex differences in specific diagnoses, such as scoliosis, become more apparent, diagnostic decisions may be more often informed by symptom history and functional impact. However, because many older children present with fluctuating symptoms in the absence of structural abnormalities [26], non-specific diagnoses remain common despite underlying sex differences in some conditions. Previous literature has also emphasised the complexity and uncertainty involved in diagnostic classification of paediatric musculoskeletal presentations [27]. Mason et al. reported similar patterns in a large UK primary care cohort, where non-specific diagnoses accounted for the majority of pediatric back pain presentations. While direct comparison is limited by differences in healthcare setting and diagnostic coding practices, the overall predominance of non-specific diagnoses observed across both studies suggests that this pattern is not unique to Danish hospital populations. Spinal pain becomes more prevalent and recurrent with age. Thus, clinicians may be less likely to identify discrete pathological causes, increasing reliance on non-specific diagnostic categories once serious pathology has been excluded. These developmental and clinical factors likely contribute to the shift from specific diagnoses in early childhood to predominantly non-specific diagnoses in adolescence.

Sex differences in diagnostic classification were minor. Female sex was associated with slightly higher odds of receiving a specific diagnosis, but the magnitude of this association was small. This finding is somewhat unexpected given that certain structural spinal conditions, particularly adolescent idiopathic scoliosis, are more prevalent in females and are associated with a higher risk of progression and need for specialist management [28, 29]. One possible explanation is that scoliosis represents only a subset of the specific diagnostic category, which also includes conditions without known sex predilection, thereby diluting the overall sex effect. However, the limited effect size suggests that diagnostic practice in hospital-based settings is largely similar for females and males once age and temporal factors are considered.

Parental or legal guardian spine history showed a modest association which diagnostic classification, with children of affected parents more likely to receive a non-specific diagnosis. This could be explained by several mechanisms. For example, parental spine-related conditions could be associated with differences in healthcare utilisation, symptom interpretation, or referral pathways. Clinicians may also interpret symptoms within a familial behavioural or psychosocial context, lowering the threshold for assigning non-specific codes. Parental pain has been shown to influence children’s symptom expression, pain sensitivity, and healthcare utilisation [17, 18]. While these interpretations remain speculative, they are consistent with existing literature on familial influences on pain and health behaviour.

We additionally observed differences in children’s diagnostic classification according to parental diagnosis type. Children with only non-specific parental spinal diagnoses more frequently received non-specific diagnoses themselves, whereas children without parental spinal diagnoses had the highest proportion of specific diagnoses. Although exploratory, these findings may reflect differences in healthcare utilisation, symptom interpretation, referral pathways, or diagnostic practices within families or even genetics. However, the mechanisms underlying these patterns cannot be determined from registry data alone.

Comorbidity burden was only weakly associated with diagnostic specificity. Higher comorbidity burden slightly increased the odds of receiving a specific diagnosis, though this pattern should be interpreted cautiously. In younger children, multiple comorbidities may signal meaningful health complexity prompting more thorough evaluation and thereby increasing the likelihood of assigning a specific spine-related diagnosis, whereas in older adolescents a higher comorbidity count may reflect accumulated transient or episodic diagnoses over time, rather than meaningful health complexity. This distinction is important and highlights a limitation in chapter-based comorbidity measures where higher comorbidity burden may reflect health care exposure, rather than disease burden.

### Clinical implications

Children’s characteristics in diagnostic classification differed across age groups, parental spine history, and comorbidity burden, although the mechanisms underlying these patterns remain uncertain. Still, whether these patterns reflect true differences in underlying pathology, differences in how symptoms evolve with development, or clinician decision-making influenced by contextual factors, cannot be determined from registry data alone. Observed associations may reflect multiple underlying clinical, behavioural, genetics, or healthcare-system factors that cannot be disentangled within the present study design [30]. The sex of the child did not impact the relationship between specific and non-specific diagnoses.

If clinician-related variation driven by diagnostic uncertainty contributes, then clearer diagnostic criteria, structured paediatric assessment pathways, and developmental guidance for evaluating spinal pain may help reduce unwarranted variability. The high prevalence of non-specific diagnoses in children and adolescents raises the question of whether some of these presentations could potentially be managed within primary care settings rather than hospital-based care. In line with adult evidence, non-specific spinal pain is typically addressed through conservative management pathways, with specialist assessment reserved for cases with features suggestive of underlying pathology [31]. As well, the high prevalence of specific diagnoses in early childhood raises the question of potential over-investigation and over-medicalisation driven by diagnostic uncertainty. Both highlighting the need for clearer age-appropriate diagnostic strategies for spinal pain.

### Strengths, limitations and future directions

This study benefits from nationwide coverage, a large sample size, and robust linkage between children and parents, providing a comprehensive overview of hospital-based diagnostic patterns for paediatric spine conditions in Denmark.

A limitation is the reliance on registry-based diagnostic codes, which reflect administrative coding rather than detailed clinical assessment. As such, misclassification bias is possible. In addition, differences in healthcare-seeking behaviour and referral patterns across population subgroups may also have influenced the observed diagnostic distributions. We also did not include socioeconomic characteristics such as parental education or household income, which may influence healthcare utilisation, referral pathways, and diagnostic processes.

In addition, analyses were restricted to index hospital diagnoses and did not capture diagnostic trajectories over time. Some children initially coded with non-specific spinal pain may later receive a more specific diagnosis following further investigation, while others may continue to be managed conservatively without reclassification.

Future research should incorporate longitudinal follow-up to examine diagnostic stability and transitions over time, particularly for high-frequency diagnoses prone to misclassification. Studies integrating clinical findings, imaging results, and patient-reported outcomes would further strengthen understanding of how diagnostic decisions are made and how they evolve across childhood and adolescence. Furthermore, future studies should examine whether specific comorbidity profiles, rather than overall burden alone, are associated with patterns in diagnostic classification.

## Conclusion

In this nationwide cohort study of nearly 50,000 Danish children, specific spine-related diagnoses were far more common in very young children, while the majority of older children received non-specific spine-related diagnoses. Diagnostic classification differed modestly according to parental spine history, comorbidity burden, and, to a lesser extent, sex. These patterns may reflect differences in clinical presentation, healthcare utilisation, referral pathways, or other unmeasured factors that could not be assessed within the present study. The predominance of non-specific diagnoses in older children indicates that many of these presentations may be more appropriately handled in primary care settings. Future research should integrate clinical examination findings, longitudinal follow-up, and patient- and clinician-level factors to better understand diagnostic pathways in paediatric spine care.

## Supplementary Information

Below is the link to the electronic supplementary material.


Supplementary Material 1



Supplementary Material 2



Supplementary Material 3



Supplementary Material 4



Supplementary Material 5



Supplementary Material 6


## Data Availability

The data that support the findings of this study are available from Statistics Denmark but restrictions apply to the availability of these data, which were used under license for the current study and are not publicly available. Data are, however, available from the authors upon reasonable request and with permission from Statistics Denmark.

## Abbreviations

CPR: Central person register
CRS: Danish civil registration system
DNPR: Danish National Patient Register
DST: Statistics Denmark
GP: General practitioner
ICD-10: International Classification of Diseases, 10th revision
IQR: Interquartile range
IR: Incidence rate
RECORD: REporting of studies Conducted using Observational Routinely-collected health Data
STROBE: Strengthening the Reporting of Observational Studies in Epidemiology
