# Supplementary material for: Specific versus non-specific hospital-based spine diagnoses in children and adolescents: a nationwide register-based cohort study
Source: Chiropr Man Therap. 2026 Jul 1;34:41. doi: 10.1186/s12998-026-00661-z (PMC13525669; doi:10.1186/s12998-026-00661-z)
Supplement: Supplementary file 1 [file 12998_2026_661_MOESM2_ESM.docx]

Supplementary Table S2. Post-hoc distribution of selected specific diagnoses by age group and sex

| **Diagnosis category** | **Age group** | **Female, n** | **Male, n** | **Total, n** |
| --- | --- | --- | --- | --- |
| All specific diagnoses | 0-4 years | 1,754 | 1,678 | 3,432 |
| All specific diagnoses | 5-17 years | 9,809 | 7,832 | 14,209 |
| Scoliosis (M41*) | 0-4 years | 304 | 313 | 617 |
| Scoliosis (M41*) | 5-17 years | 7,149 | 4,633 | 11,782 |

*Scoliosis defined as ICD-10 codes M41*. Counts represent index hospital-based diagnoses. Age groups defined as 0–4 and 5–17 years.*
