# Supplementary material for: Specific versus non-specific hospital-based spine diagnoses in children and adolescents: a nationwide register-based cohort study
Source: Chiropr Man Therap. 2026 Jul 1;34:41. doi: 10.1186/s12998-026-00661-z (PMC13525669; doi:10.1186/s12998-026-00661-z)
Supplement: Supplementary file 2 [file 12998_2026_661_MOESM3_ESM.docx]

**RECORD checklist**

**TITLE AND ABSTRACT**

| **Item** | **Requirement** | **Page** |
| --- | --- | --- |
| 1.1 | Data type specified | p.1 |
| 1.2 | Region + timeframe | p.1 |
| 1.3 | Data linkage stated | p.4–5 |

**INTRODUCTION**

| **Item** | **Requirement** | **Page** |
| --- | --- | --- |
| 2 | Background | p.2–3 |
| 3 | Objectives | p.3 |

**METHODS**

**Study design & setting**

| **Item** | **Requirement** | **Page** |
| --- | --- | --- |
| 4 | Study design | p.4 |
| 5 | Setting | p.4–5 |

**Participants**

| **Item** | **Requirement** | **Page** |
| --- | --- | --- |
| 6.1 | Codes/algorithms for population | p.5–6 |
| 6.2 | Validation of codes | - |
| 6.3 | Linkage flow | - |

**Variables**

| **Item** | **Requirement** | **Page** |
| --- | --- | --- |
| 7.1 | Complete code list | p.6 + suppl |

**Data sources / bias**

| **Item** | **Requirement** | **Page** |
| --- | --- | --- |
| 8 | Data sources | p.4–5 |
| 9 | Bias | p.7 |

**Statistical methods**

| **Item** | **Requirement** | **Page** |
| --- | --- | --- |
| 12 | Statistical methods | p.7 |

**Data access & cleaning (RECORD-specifik)**

| **Item** | **Requirement** | **Page** |
| --- | --- | --- |
| 12.1 | Data access | p.5 |
| 12.2 | Data cleaning | p.7 |

**Linkage**

| **Item** | **Requirement** | **Page** |
| --- | --- | --- |
| 12.3 | Linkage method | p.6 |

**RESULTS**

**Participants**

| **Item** | **Requirement** | **Page** |
| --- | --- | --- |
| 13 | Selection of population | p.5 + 7 |
| 13.1 | Detailed selection | p.5 |

**Descriptive data**

| **Item** | **Requirement** | **Page** |
| --- | --- | --- |
| 14 | Descriptive data | p.7–8 |

**Outcomes & results**

| **Item** | **Requirement** | **Page** |
| --- | --- | --- |
| 15 | Outcome data | p.8 |
| 16 | Main results | p.9 |
| 17 | Other analyses | p.9 |

**DISCUSSION**

| **Item** | **Requirement** | **Page** |
| --- | --- | --- |
| 18 | Key results | p.10 |
| 19 | Limitations | p.12 |
| 19.1 | Registry-specific limitations | p.12 |
| 20 | Interpretation | p.10–12 |
| 21 | Generalisability | p.12 |

**OTHER INFORMATION**

| **Item** | **Requirement** | **Page** |
| --- | --- | --- |
| 22 | Funding | p.13 |
| 22.1 | Data/code access | p.13 |
