# Supplementary material for: Specific versus non-specific hospital-based spine diagnoses in children and adolescents: a nationwide register-based cohort study
Source: Chiropr Man Therap. 2026 Jul 1;34:41. doi: 10.1186/s12998-026-00661-z (PMC13525669; doi:10.1186/s12998-026-00661-z)
Supplement: Supplementary file 3 [file 12998_2026_661_MOESM4_ESM.docx]

**STROBE checklist (cohort)**

**TITLE AND ABSTRACT**

| **Item** | **Recommendation** | **Page** |
| --- | --- | --- |
| 1a | Indicate study design in title/abstract | p.1 |
| 1b | Informative abstract | p.1 |

**INTRODUCTION**

| **Item** | **Recommendation** | **Page** |
| --- | --- | --- |
| 2 | Background/rationale | p.2-3 |
| 3 | Objectives | p.3 |

**METHODS**

| **Item** | **Recommendation** | **Page** |
| --- | --- | --- |
| 4 | Study design | p.4 |
| 5 | Setting | p.4-5 |
| 6a | Participants | p.5 |
| 6b | Matching | NA |
| 7 | Variables | p.5-6 |
| 8 | Data sources/measurement | p.4-5 |
| 9 | Bias | p.7 |
| 10 | Study size | p.5 |
| 11 | Quantitative variables | p.6 |
| 12a | Statistical methods | p.7 |
| 12b | Subgroups/interactions | p.7 |
| 12c | Missing data | p. 8 |
| 12d | Loss to follow-up | NA |
| 12e | Sensitivity analyses | - |

**RESULTS**

| **Item** | **Recommendation** | **Page** |
| --- | --- | --- |
| 13a | Participant flow | p.7 |
| 13b | Non-participation | NA |
| 13c | Flow diagram | - |
| 14a | Descriptive data | p.7-8 |
| 14b | Missing data | p. 8 |
| 14c | Follow-up time | NA |
| 15 | Outcome data | p.8 |
| 16a | Main results | p.9 |
| 16b | Category boundaries | p.6 |
| 16c | Absolute risk | NA |
| 17 | Other analyses | p.9 |

**DISCUSSION**

| **Item** | **Recommendation** | **Page** |
| --- | --- | --- |
| 18 | Key results | p.10 |
| 19 | Limitations | p.12 |
| 20 | Interpretation | p.10-12 |
| 21 | Generalisability | p.12 |

**OTHER INFORMATION**

| **Item** | **Recommendation** | **Page** |
| --- | --- | --- |
| 22 | Funding | p.13 |
