# Supplementary material for: Specific versus non-specific hospital-based spine diagnoses in children and adolescents: a nationwide register-based cohort study
Source: Chiropr Man Therap. 2026 Jul 1;34:41. doi: 10.1186/s12998-026-00661-z (PMC13525669; doi:10.1186/s12998-026-00661-z)
Supplement: Supplementary file 5 [file 12998_2026_661_MOESM6_ESM.docx]

The distribution of children’s diagnosis type based on parental diagnosis groups.

| **Parental diagnosis type** | **Child non-specific n (%)** | **Child specific n (%)** |
| --- | --- | --- |
| No parental spinal diagnosis | 21,866 (62.7) | 12,998 (37.3) |
| Only non-specific parental diagnosis | 5,162 (70.5) | 2,158 (29.5) |
| Any specific parental diagnosis | 4,962 (66.9) | 2,450 (33.1) |
